# Inappropriate cadherin switching in the mouse epiblast compromises proper signaling between the epiblast and the extraembryonic ectoderm during gastrulation

**DOI:** 10.1038/srep26562

**Published:** 2016-05-24

**Authors:** M. Felicia Basilicata, Marcus Frank, Davor Solter, Thomas Brabletz, Marc P. Stemmler

**Affiliations:** 1 Department of Molecular Embryology, Max-Planck Institute of Immunobiology and Epigenetics, Stübeweg 51, 79108 Freiburg, Germany; 2 Electron Microscopy Center, University Medicine Rostock, Strempelstr. 14, 18057 Rostock, Germany; 3 Epithelial Epigenetics and Development Lab, Institute of Medical Biology, A^*^STAR, Singapore; 4 Department of Experimental Medicine I, Nikolaus-Fiebiger Center for Molecular Medicine, University of Erlangen-Nürnberg, Glückstr. 6, 91054 Erlangen, Germany

## Abstract

Cadherin switching from E-cadherin (E-cad) to N-cadherin (N-cad) is a key step of the epithelial-mesenchymal transition (EMT) processes that occurs during gastrulation and cancer progression. We investigate whether cadherins actively participate in progression of EMT by crosstalk to signaling pathways. We apply ectopic cadherin switching before the onset of mouse gastrulation. Mutants with an induced E-cad to N-cad switch (Ncadki) die around E8.5. Severe morphological changes including a small epiblast, a rounded shape, an enlarged extra-embryonic compartment and lack of the amnion, combined with a massive cell detachment from the ectodermal layer are detected. In contrast to epiblast-specific E-cad depletion, gastrulation is initiated in Ncadki embryos, but patterning of the germ-layers is abnormal. An overall reduction in BMP signaling, expansion of *Nodal* and *Eomes* domains, combined with reduced *Wnt3a* expression at the primitive streak is observed. Our results show that in addition to cadherin-dependent adhesion, proper embryonic development requires E-cad mediated signaling function to facilitate a feedback loop that stabilizes *Bmp4* and *Bmp2* expression in the extraembryonic ectoderm and sustained downstream activity in the epiblast. Moreover, for proper morphogenesis a fine-tuned spatio-temporal control of cadherin switching is required during EMT at gastrulation to avoid premature cell detachment and migration.

The “classical cadherins”, E- and N-cadherin play pivotal roles in the proper formation of a mammalian embryo and in the maintenance of tissue homeostasis, by dynamically regulating cell-cell adhesion. Disruption of E-cadherin (*Cdh1*) gene function is affecting morula compaction and blastocyst formation^1^,^2^ and is crucial for epithelial cell function^3^,^4^. N-cadherin is first expressed in the emerging mesoderm cells during gastrulation and N-cadherin deficient embryos suffer from patterning defects of the axial mesoderm and malformation of the heart and the brain^5^. Accumulating reports suggest that cadherins are not only involved in mediating cell adhesion, but also participate in the modulation of signaling cascades crucial for embryogenesis, e.g. with growth factor receptors like Lifr and Igf1r to maintain pluripotency in embryonic stem cells (ESCs) and to facilitate cell survival cues in the trophectoderm^2^,^6^,^7^,^8^. Importantly, N-cad can substitute for deficient adhesion, but not for signaling, since it is incapable in forming complexes with the same RTKs as E-cad. Moreover, the individual contribution of cadherins in signaling is also reflected in breast cancer cell lines. Here, interaction of FGFR1 and N-cad attenuates or stimulates FGF signaling combined with alterations in cell adhesion^9^,^10^,^11^ and promotes epithelial-mesenchymal transition (EMT)^10^,^12^, whereas EGFR and E-cad ligation leads to EGFR activation and disruption of cell adhesion^13^,^14^.

During mammalian development as well as during cancer progression EMT plays a crucial role to enable cell migration and change cell phenotypes and characteristics. In the cup shaped egg cylinder of a mouse embryo at E6.5, complex signaling cascades and cell movements allow the formation of the posterior primitive streak (PS) and the mesoderm. E-cad positive cells of the ectoderm lose their apical-basal polarity and cell-cell contacts to ingress the PS. They lose *E-cad* expression and gain expression of *N-cad* combined with a more spindle shape morphology, a front-rear polarity and a motile phenotype. Blocking EMT and *E-cad* downregulation, e.g. in *Snai1* mutant embryos, the mesoderm does not form and cells are clumped at the PS^15^. Cells in a solid tumor often hijack this program to support cell dissemination and invasion. Complex mechanisms and networks are required to induce the profound changes in cellular architecture, gene expression patterns and switching in cadherin expression during EMT^16^. Although the cadherin switch is a hallmark of EMT the role of N-cad in the process of gastrulation and mesoderm migration is not well understood. Whether the switch is just a consequence of the demands of the morphogenetic program or whether cadherins also actively participate in signaling cues to drive EMT progression remains elusive.

Here, we analyzed the unique properties of cadherins and whether a forced switch of cadherin expression is affecting signaling in the pregastrulating mouse embryo. We found that E-cad is crucial for proper morphogenesis and cell movements during gastrulation. Embryos that express *N-cad* show altered BMP activity, resulting in an atrophied epiblast, inappropriate loss of cells into the amniotic cavity and inefficient patterning of the extraembryonic mesoderm, indicating that E-cad and a tight spatio-temporal regulation of cadherin switching is indispensable for maintaining signaling loops between embryonic and extraembryonic tissues for establishing proper BMP signaling cues during axis specification.

## Results

### Ncadki embryos die at E8.5 due to growth retardation and a degenerated epiblast

In order to experimentally control the cadherin switch isolated from a complete EMT program, we made use of a gene replacement approach. We induced the cadherin switch prior to the onset of gastrulation combining an Ncadki allele (*N-cad* cDNA expressed under the control of the *E-cad* locus) and the conditional *E-cad* knockout allele (Ecad^fl^). Recombination and the switch were restricted to the epiblast during implantation by the use of Sox2Cre^17^. Mutant Ncadki (Ecad^Ncad/ΔEpi^;Sox2Cre) and control progeny (Ecad^Ncad/fl^, Ecad^+/fl^ and Ecad^+/ΔEpi^;Sox2Cre) were analyzed in comparison to epiblast-specific *E-cad* deficient embryos (Ecadnull; Ecad^−/ΔEpi^;Sox2Cre) (Fig. S1A). Efficient and completed recombination was observed earlier than E6.5 using the Rosa26R allele (Fig. S1B) and anti-E-cad imunolabeling. Only few patches of E-cad positive cells with a more apical localization were observed at E5.5 anticipating the complete loss at E6.5 and E7.5 (Fig. S1C). The N-cad staining of the epiblast of the mutants showed a similar distribution as E-cad in controls, whereas neither E-cad nor N-cad staining was detected in epiblasts of Ecadnull embryos (Fig. S1C).

**Figure 1:**
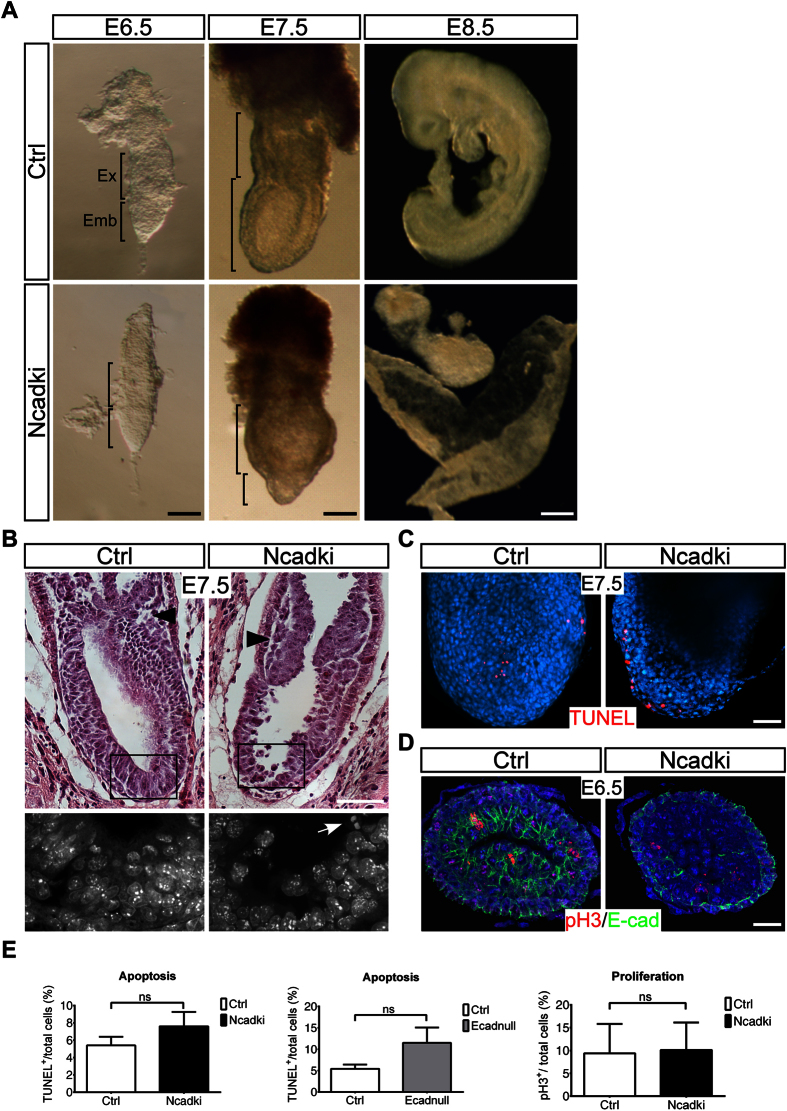
Ectopic switching from E-cad to N-cad expression in the epiblast before gastrulation results in degeneration of the epiblast at E7.5 and embryos are incapable to survive beyond E8.5. (**A**) Bright field images of Ecad^Ncad/ΔEpi^ (Ncadki) mutant and Ecad^Ncad/fl^ and Ecad^+/ΔEpi^ control (Ctrl) embryos at stages between E6.5 and E8.5, showing size reduction of the epiblast in mutant embryos at E7.5. Scale bars represent 100 μm for left and middle and 250 μm for right panel. (**B**) Histological analysis of sagittal sections at E7.5. In Ncadki embryos the absence of morphological borders between germ-layers are apparent. DAPI staining of the boxed region shows normal distribution of chromatin in detaching cells (lower panel). Arrow indicates condensed chromatin of a pyknotic nucleus. Note, that mutants fail to form and fuse the amnion (arrowhead). Scale bar represents 100 μm (**C,D**) Analysis of apoptosis at E7.5 by TUNEL (**C**) and proliferation at E6.5 by pH3 staining (**D**) with only minor differences between mutant and control embryos. TUNEL-positive (**C**) and pH3-positive cells (**D**) are shown in red and E-cad in green (**D**), nuclei are stained with DAPI. Scale bar represents 50 μm for TUNEL and 20 μm for pH3 staining. Embryos are presented with proximal to the top and anterior to the left (E6.5-E7.5) and anterior to the top and dorsal to the right (E8.5). (**E**) Quantification of the analysis shown in (**C,D**), including Ecad^−/ΔEpi^ embryos (Ecadnull); n = 5. Spots were counted using ImageJ multicounting function.

Embryos between E6.0 and E8.5 were isolated in normal Mendelian ratios (not shown). Until E6.5 embryos of all genotypes were morphologically indistinguishable (Fig. 1A, Fig. S1D). However, at the late streak stage (E7.0-E7.5) mutant embryos showed a severe phenotype with embryonic parts being largely reduced in size compared to control embryos (Fig. 1A, Fig. S1D). Ncadki embryos were lethal at E8.5 when only unstructured clumps of cells of embryonic origin were identified in the normally growing yolk sac (Fig. 1A). Very strikingly, although the severity of the phenotype varied (Fig. S2A), all mutant embryos showed defects in amnion formation at E7.5 (Fig. 1B, arrowheads). In contrast, Ecadnull embryos were consistently smaller already at E6.5, showing defects in the embryonic and the extraembryonic parts (Figs S1C and S2B) and increased apoptosis at E7.0 (Fig. 1C, Fig. S2C), pointing to a more severe phenotype than that of Ncadki embryos.

These observations showed that ectopic cadherin switching in the epiblast before the onset of gastrulation in Ncadki embryos results in severe morphogenetic defects incompatible with successful embryogenesis.

### In Ncadki embryos cells are detaching from the epiblast layer and accumulate in the proamniotic cavity

Hematoxylin and eosin (H&E) staining of embryonic tissue sections between E7.0 and E7.5 revealed a general impairment of the embryonic and cellular architecture. In control embryos ectoderm, mesoderm and endoderm layers are characterized by distinct cellular and nuclear shapes, defining discrete borders between each layer. This architecture appeared completely lost in Ncadki embryos resulting in cell intermingling that did not allow discrimination of the individual germ-layers on H&E stained sections (Fig. 1B). Interestingly, cells were also found in clumps or as singlets in the proamniotic cavity. High resolution transmission electron micrographs of this area in mutant embryos indicated that the cells have been excluded from the ectoderm by a process reminiscent of cell delamination or premature EMT (Fig. S2D,E). Cells in the initial phase of extrusion and still in vicinity of the epiblast did not show signs of pyknotic DNA, but normal non-condensed distribution of chromatin (Fig. 1B), and therefore were not considered to be necrotic or apoptotic. Cell viability was then lost when the cells were released into the proamniotic cavity, with increased condensed chromatin (Fig. 1B, arrow) and TUNEL labeling (data not shown).

The cadherin-switch embryos showed comparable amounts of apoptotic cells as controls, distributed throughout all embryonic layers (Fig. 1C,E). Apoptosis was more a consequence of cell detachment and not directly induced by cadherin switching as observed during preimplantation development^8^. Similarly, BrdU labeling, PCNA, Ki67 (data not shown) and pH3 detection (for analysis of mitotic events) did not reveal significant changes in proliferation of the mutant epiblast at E6.5 (Fig. 1D,E) and E7.5 (data not shown).

Taken together, the small size of the Ncadki embryo led to a fatal collapse of the embryonic program. Degeneration of the epiblast was not due to alterations in proliferation or apoptosis rates but rather a consequence of substantial cell loss that led to a premature exhaustion of pluripotent cells within the epiblast.

### Gastrulation is initiated independent of the ectopic cadherin switch and all germ-layers retain their differentiation capacities in Ncadki embryos

**Figure 2:**
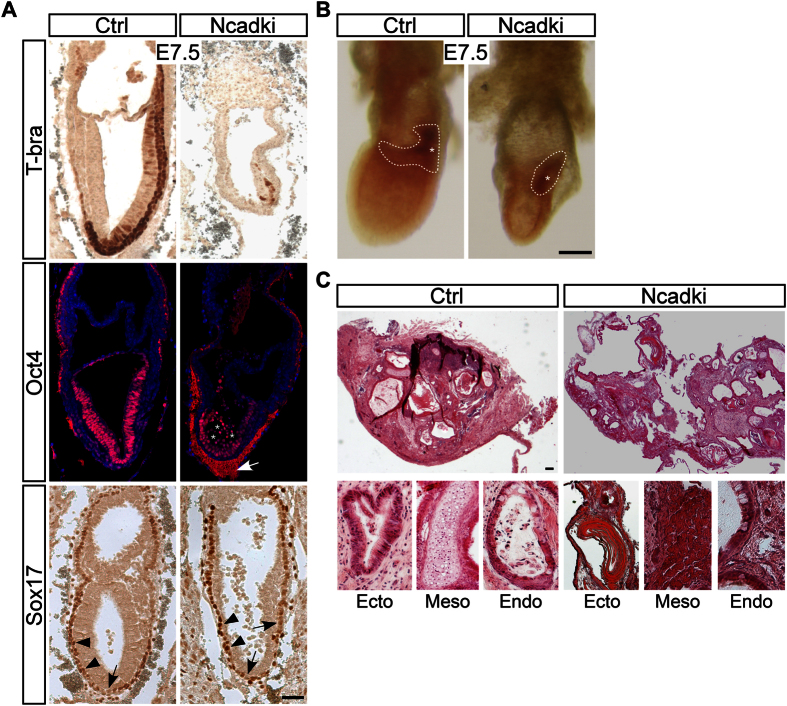
Ncadki embryos properly initiate gastrulation and cells retain their full differentiation capacity. (**A**) Immunohistochemistry and immunofluorescence labeling of key markers of ectoderm (Oct4), mesoderm (T-brachyury, T-Bra) and DE (Sox17). Asterisks indicate Oct4-positive detaching cells, arrowheads highlight the intercalating DE cells with increased Sox17 expression and arrows the VE cells. Note that the cell free area between the Reichert’s membrane and VE of Ncadki embryos shows high amounts of unspecific staining for antibodies raised in mice (arrow). Scale bar represents 50 μm. (**B**) Alkaline phosphatase staining to detect primordial germ cells (PGCs). White dashed lines indicate the area of PGCs which is present but smaller in Ncadki embryos. Embryos are presented with proximal to the top and anterior to the left. Asterisks highlight the PS spot of germ-cell formation. Scale bar represents 100 μm. (**C**) Overview of H&E stained sections of teratomas generated by transplantation under the kidney capsule with examples of derivatives of ectodermal, mesodermal and endodermal origin in higher magnification. Scale bar represents 50 μm.

The continuous cell detachment of Ncadki mutants may be due to a premature initiation of EMT and gastrulation. We analyzed specific markers of the three germ layers at E7.5. In Ncadki embryos pluripotent cells of the ectoderm were identified by Oct4 staining showing a similar staining as controls in addition to the Oct4-positive delaminating cells, indicating that the detaching cells originate mainly from the ectoderm (Fig. 2A, middle panel, asterisks). In normal embryos T-bra is expressed in the PS and highlights mesoderm induction. The anti-T-bra antibody specifically stained a subset of cells confined to one side of the embryo in controls and in the mutants. In Ncadki embryos the amount of cells was largely reduced and restricted to the embryonic part, but not found at ectopic sites or in detaching cells. T-bra positive cells of control embryos extended posteriorly along the proximal-distal (P-D) axis including the extraembryonic mesoderm (Fig. 2A, upper panel). Sox17 was used to identify the definitive endoderm (DE) cells^18^ that intercalate with increased Sox17 expression levels into the visceral endoderm (VE)^19^. In Ncadki and in control embryos Sox17 expression was low and restricted to the primitive endoderm at E6.5. Cells in a characteristic “salt and pepper“ pattern with higher (DE) and lower Sox17 expression (VE)^20^,^21^ were identified in control and mutant embryos without striking differences (Fig. 2A, lower panel, arrowheads, arrows). In addition, alkaline phosphatase staining on whole mount embryos revealed proper specification of primordial germ cells (PGCs), however in reduced amounts (Fig. 2B), indicating unaffected initial specification of the three germ layers in the mutant embryos.

We induced teratoma formation by transplanting the embryonic parts of Ncadki and control embryos under the kidney capsule of syngenic mice to analyze the full differentiation capacities of mutant cells. After eight weeks teratomas were forming independent of the genotype (Fig. 2C) with comparable sizes but slight differences in texture and rigidity (data not shown). We observed that teratomas from Ncadki mutant embryos were more attached to the host tissue of the kidney, but none of them showed a clear invasive phenotype. Histological analysis of teratomas revealed that Ncadki and control embryos formed the same variety of tissues. Ncadki-derived teratomas also contained tissue derivatives of ectodermal, endodermal and mesodermal origin (Fig. 2C), for instance neuroepithelium, muscle, cartilage and intestinal epithelium^22^,^23^,^24^. No E-cad labeling was detected in any of the Ncadki specimens and N-cad was present on the cell membranes of epithelial structures, resembling the staining of E-cad in controls in addition to the neuroectoderm and muscle tissues that expressed endogenous N-cad (Fig. S3). Not surprisingly these findings are in agreement with previous results in embryonic stem cells^25^.

Our results show that although the size of the mutant epiblast is reduced, all signaling components to initiate gastrulation at one side of the embryo are present and cells seem to properly respond to the signaling cues. However, only a few mesoderm cells arise which reside exclusively in the embryonic part. Despite the severe phenotype all three germ-layers retain the potential to differentiate into a large variety of tissues.

### Ectopic N-cad expression alters mesodermal cell shape

**Figure 3:**
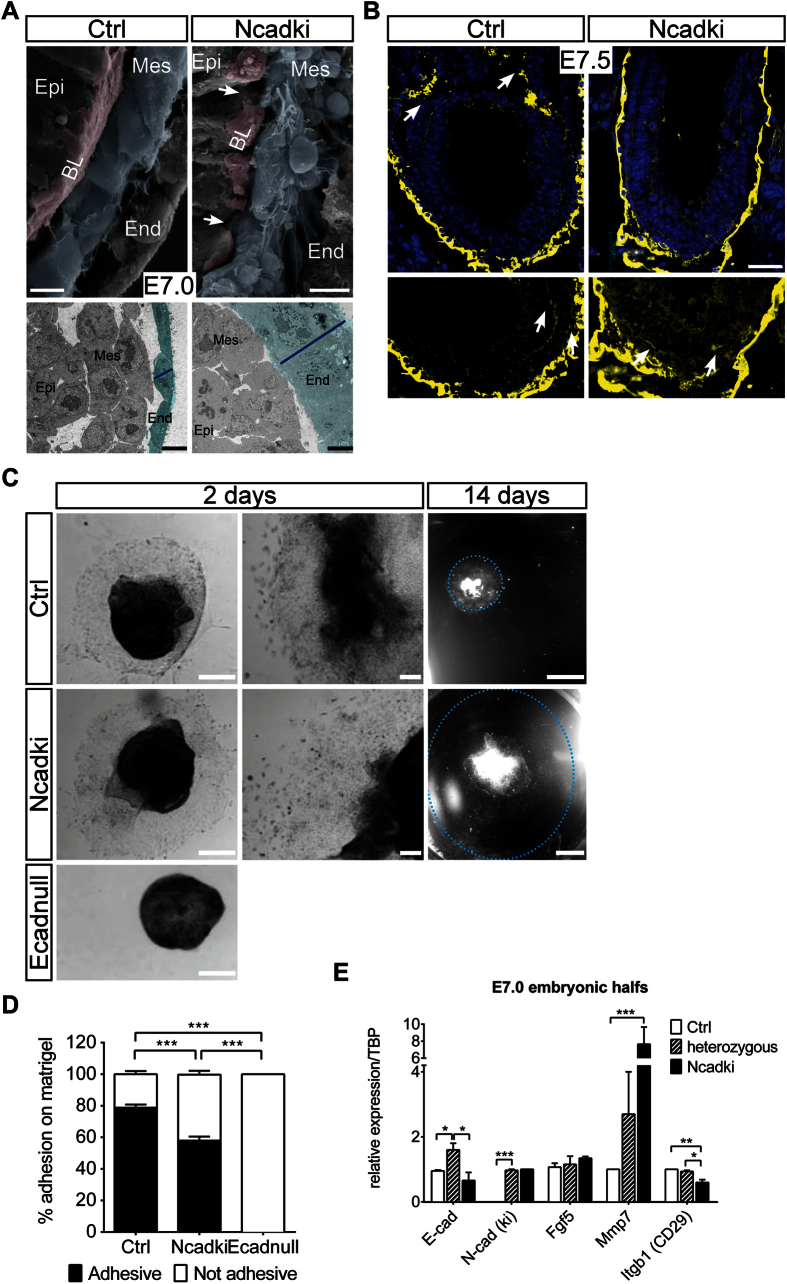
Tissue architecture and extracellular matrix components are changing upon ectopic cadherin switching. (**A**) Scanning electron microscopy shows loss of the smooth surface and attached basal lamina at mutant ectodermal cells (purple layer, arrows) as well as malformed mesoderm cells (blue) compared to controls. An expansion of the radial dimension of the VE cells (green) is seen in the mutant e.g. at the primitive streak region with transmission electron microscopy (lower panel) at E7.0 (blue line). Epi, epiblast; Mes, mesoderm; End, endoderm; BL, basal lamina. Scale bars represent 5 μm. (**B**) Anti-Laminin staining (yellow) and counterstaining of nuclei with DAPI (blue) reveals a loss of Laminin in the extraembryonic part at the region of the amnion in E7.5 Ncadki embryos that is found in control embryos (arrows in upper panel). No difference is detected in the gradual reduction in Laminin distribution in the embryonic part from proximal to distal, as well as in the reduction of Laminin between ectoderm and mesoderm at the side of the primitive streak in mutants and controls (arrows in lower panel). Scale bar represents 50 μm. (**C**) Functional analysis of the ECM of Ncadki embryos by plating explants of embryonic halfs to matrigel. Ecadnull embryos fail to attach and spread, whereas Ncadki (4/7) and control embryos (34/42) succeed. Explants of Ncadki spread much faster and cover the whole plate within 14 days (dashed line). Scale bars represent 250 μm for left, 100 μm for middle and 500 μm for right panels. (**D**) Quantification of successful attachment to the plates of embryonic explants of indicated genotypes. (**E**) qRT-PCR analysis of Ecad^Ncad/ΔEpi^;Sox2Cre (Ncadki), Ecad^Ncad/+^;Sox2Cre (heterozygous) and Ecad^+/ΔEpi^;Sox2Cre (Ctrl) embryonic halfs, showing a >7fold increase in Mmp7 and slight reduction of β1-Integrin (CD29) expression in Ncadki. The specimen preparation does not exclusively contain epiblast cells and unrecombined VE cells are included in the RNA isolation. Accordingly, effects are likely to be less pronounced as indicated by incomplete reduction in E-cad levels.

To better understand the cellular changes that induce the degenerated epiblast phenotype we analyzed cell-matrix interaction around the time-point when changes became apparent. Ultrastructural analysis of areas with excess cell detachment into the proamniotic cavity in E7.0 mutant embryos identified that epiblast cells still form normal cell-cell contacts and apical-basal cell polarity with no striking differences in the organization of apical microvilli and adherens and tight junctions (Fig. S4A). This was confirmed by normal apical distribution of the tight junction component ZO-1 and of Ezrin (Fig. S4B,D). β-catenin (β-cat) was used as marker for adherens junction assembly and was found less intense at the membrane of the ectoderm in mutant embryos, pointing to either a weaker interaction to ectopic N-cad or to reduced expression of the Ncadki allele (Fig. S4C). High resolution analysis of cell morphology by scanning electron microscopy confirmed a comparable normal cell shape of the ectoderm layer (Fig. 3A). Nevertheless, the cells of the ectoderm were not forming a very smooth surface on the side facing the mesoderm (Fig. 3A, upper panel) and the VE cells had a much larger radial diameter in N-cadki than in control embryos (Fig. 3A lower panel, compare size of blue lines). Cells of the mesoderm were malformed with longer, non-directional fibroblast-like protrusions, most likely representing filopodia. In contrast mesoderm cells of control embryos showed a more cobblestone-like morphology with short directed filopodia. Intriguingly, the mesoderm phenotype was surprising, as after gastrulation the E-cad locus including the Ncadki allele is downregulated, indicating that these changes were adopted prior to gastrulation. In addition, a reduction in the basal lamina covering the basal epiblast cell process was detected in mutant embryos (Fig. 3A, arrows, compare continuous basal lamina in control vs. a patchy coverage in mutant embryos). Molecular analysis of the basal lamina with a pan-laminin antibody highlighted a complete loss of laminin staining at the level of the extraembryonic amniotic fold in the Ncadki embryos, whereas no clear differences were observed in the embryonic part (Fig. 3B, arrows). However, in the region of the PS in both control and mutant embryos laminin staining was correctly reduced between mesoderm and ectoderm, without indications of premature or ectopic basal lamina breakdown that could account for cell delamination and ectopic EMT in the mutants.

### Ncadki embryos show altered ECM composition and cell-matrix attachment

We wanted to further elucidate a potential defect in the crosstalk between cadherins, integrins and extracellular matrix cell adhesion, which is crucial for modulating activities of different signaling cascades in epithelia. It was shown that cadherin and integrin interaction modifies cell adhesion and cell motility of normal and tumor cells^26^,^27^,^28^. To assess whether improper extracellular matrix adhesion or degradation contributed to the phenotype, we performed embryonic explant cultures of dissected epiblasts of embryos at E7.0^29^. Interestingly, Ncadki embryos showed a reduction in ECM adhesion capability, as only 4 out of 7 (~57%) plated embryos and none of the Ecadnull epiblasts attached to matrigel-ECM, whereas 34 out of 42 control embryos (~81%) succeeded (Fig. 3C,D). Cells from Ncadki started to migrate out of the explant shortly after attachment to the matrigel, but in comparison to controls, did not form a compact rim and instead spread over the whole plate within 15 days after plating (Fig. 3C).

To understand the molecular basis for altered cadherin-ECM crosstalk in Ncadki mutants we analyzed mRNA expression of key genes from single E7.0 embryonic halfs (epiblast+VE). *E-cad* expression was not completely reduced in the mutants due to presence of VE cells (Fig. 3E), whereas Ncadki expression was only detectable in heterozygous (Ecad^Ncad/fl^) and Ncadki embryos (Ecad^Ncad/ΔEpi^;Sox2Cre). *Fgf5*, as marker for epiblast identity and potency, was not altered. By focusing on proteins that are involved in basal lamina composition and degradation we analyzed the expression of epithelial-derived matrix metalloproteases (MMPs). Upregulation of MMPs may accelerate basal lamina degradation and induce a spindle-shape and more motile phenotype of the mesoderm in Ncadki mutants. We found increased expression of *Mmp7* that was combined with a significant reduction in *β1-integrin* (*Itgb1*, CD29) mRNA levels (Fig. 3E), indicating differences in ECM composition and interaction in the mutants. Interestingly, even control embryos, carrying the Ncadki and one intact E-cad allele, that displayed no detectable phenotype, also expressed significantly higher amounts of *Mmp7* that further increased upon E-cad loss in Ncadki embryos (Fig. 3D).

These results show that the composition of the basal lamina of the mutants might have been altered due to effects on gene expression in the epiblast, together affecting cell-matrix adhesion and migration of mesoderm cells. However, whether increased *Mmp7* and decreased *β1-integrin* expression directly affects laminin composition, distribution, rigidity and function with impact also on amnion formation remains unclear.

### BMP signaling is impaired in Ncadki mutants affecting mesoderm and amnion formation

**Figure 4:**
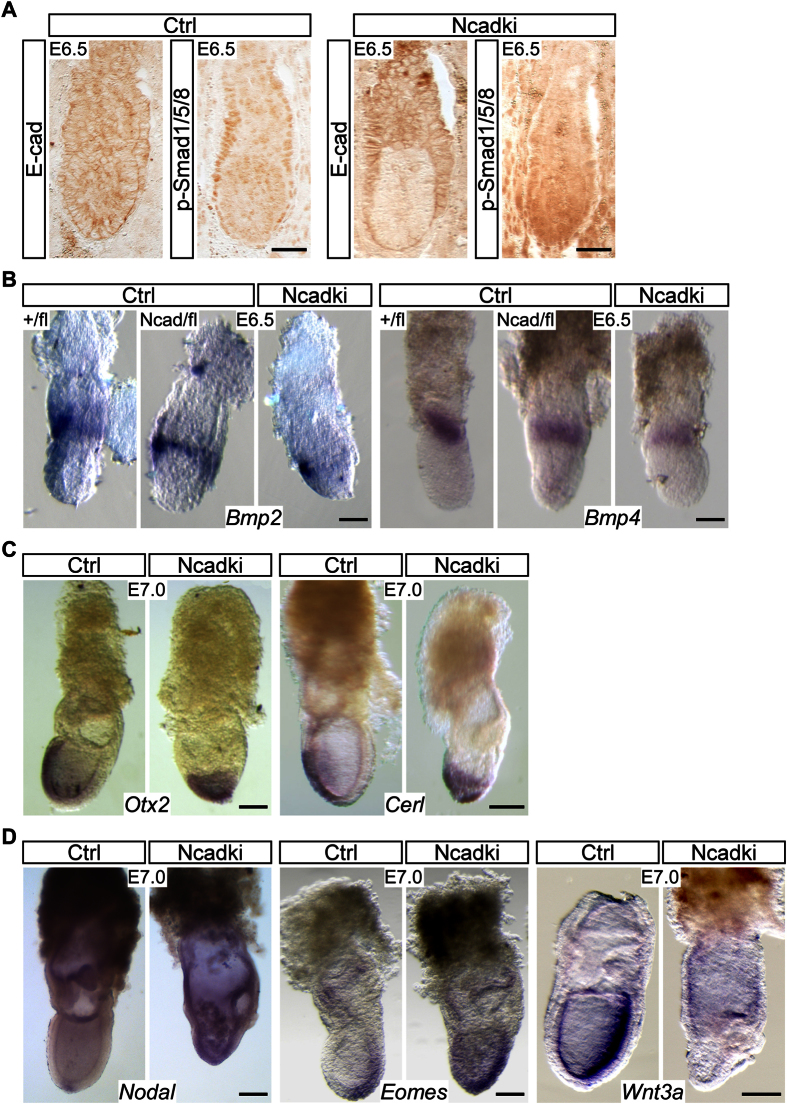
Ncadki embryos show defects in BMP signaling with altered expression of downstream signaling and effector molecules. (**A**) Anti-pSMAD1/5/8 immunohistochemistry of E6.5 embryos displays strong reduction in pSMAD1/5/8 levels in the VE of Ncadki mutants. Scale bars represent 50 μm. (**B**) Whole mount *in situ* hybridization of control (+/fl, Ecad^+/fl^ and Ncad/fl, Ecad^Ncad/fl^) and mutant embryos at E6.5 with Bmp2 (left) and Bmp4 (right) riboprobes that show a substantial reduction in mRNA levels in the mutant extraembryonic ectoderm. Scale bars represent 50 μm. (**C**) Axis specification and downstream Bmp signaling is affected in Ncadki embryos at E7.0, detected by marker genes Otx2 (left) and Cerl (right) by WISH showing that they remain localized to the distal tip of the embryo. Scale bars represent 100 μm. (**D**) Detection of mRNA of Nodal and its downstream target Eomes shows expansion of the expression domain in Ncadki embryos, whereas Wnt3a is almost absent. Embryos are orientated with proximal to the top and anterior to the left. Scale bars represent 100 μm.

To analyze the molecular basis of the defects in amnion formation we performed *in situ* hybridization of key signaling components and marker genes of gastrulating Ncadki embryos. In addition to their pivotal roles during axis specification and gastrulation, BMPs establish the key signaling cue driving amnion formation^30^,^31^,^32^,^33^,^34^. In our mutants phosphorylated Smads 1, 5 and 8 (pSmad1/5/8, active forms and common downstream effectors of BMP signaling) were reduced in the parietal endoderm and proximal VE, but found ectopically at the apical side of the egg-cylinder embryo (Fig. 4A). Bmp2 and Bmp4 are expressed in the extraembryonic ectoderm between E6.0 and E7.5 and known to signal to the embryonic part. We detected lower expression levels of both genes in mutant embryos already before morphological defects became apparent, whereas coexpression of *E-cad* and *N-cad* in heterozygous embryos (Ecad^Ncad/fl^) or deletion of one or both copies of *E-cad* alone did not affect *Bmp2* and *Bmp4* expression considerably (Fig. 4B, Fig. S5A). Downstream effects of proper BMP signaling include restriction and translocation of the *Cerl* and *Otx2* expression domains during anterior VE (AVE) migration and neuroectoderm specification^35^. In consequence of altered expression of BMPs we observed an expansion of the *Cerl* expression domain at E7.5 in Ncadki embryos. *Cerl* mRNA showed patches with stronger expression around the distal tip of the embryo, presumably due to an impaired migration of the AVE. In addition, *Otx2* showed a similar atypic pattern with a diffuse mRNA distribution over the entire embryonic part, in contrast to a limited expression in the anterior neuroectoderm of control embryos (Fig. 4C). *Nodal*, another member of the TGFβ superfamily is ubiquitously expressed in the entire epiblast between E4.5-E6.5 and becomes restricted to the PS region shortly after E6.5 and later to the node. In contrast to control embryos *Nodal* mRNA was found homogeneously distributed in the extraembryonic ectoderm (EXE) and the epiblast of Ncadki mutant embryos at E7.5 (Fig. 4D). Consequently, the expression domain of its downstream target and mesoderm marker *Eomes* increased towards the anterior part of mutant embryos (Fig. 4D). Most likely the residual BMP activity in the EXE led to inefficient activation of *Wnt3* and *Wnt3a* expression in the posterior part of mutant embryos at E7.5 (Fig. 4D). It remains unclear whether the residual Wnt3a activity or induction by other signaling pathways is activating *T-Bra* expression in the mutant embryos. Of note, the residual Bmp4 and Bmp2 were sufficient to induce PGCs, but to a lesser extent, comparable to reduced BMP signaling in *Bmp4*^+/−^ embryos^33^.

Our results confirm a substantial defect in *Bmp2* and *Bmp4* expression and downstream signaling, affecting marker genes involved in axis specification and patterning.

### The lethal phenotype of cadherin-switch mutants is a result of defects in multifactorial traits involving both extraembryonic and embryonic parts

**Figure 5:**
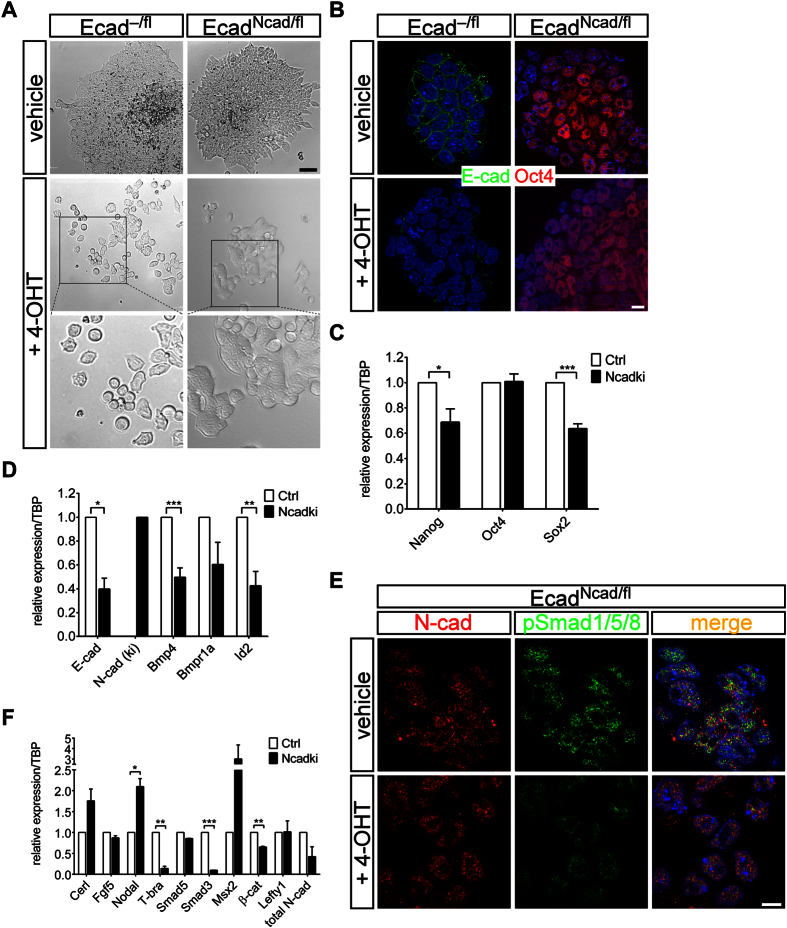
Isolated EpiSCs from Ncadki show reduced BMP signaling resulting in changes in gene expression. (**A**) Established Ecad^Ncad/fl^ EpiSCs transfected with pCAGGS-CreERT2 show characteristic colony morphology in the absence of 4-OH-tamoxifen (vehicle). Upon induction of recombination by application of tamoxifen (+4-OHT) Ecad^Ncad/Δ^ cells show a slightly different colony morphology, but still form compact colonies. In contrast, Ecad^−/Δ^ cells lose cell-cell contacts and grow as single cells. Scale bar represents 50 μm. (**B**,**C**) qRT-PCR analysis (**B**) and immunofluorescence staining (**C**) of pluripotency marker genes show a reduction of Nanog and Sox2 levels to 80%, but constant Oct4 expression in Ecad^Ncad/Δ^ (Ncadki) in comparison to Ecad^+/Δ^ or Ecad^−/fl^ (Ctrl) EpiSCs. qPCR was normalized to TBP and represented relative to controls (vehicle-treated). Scale bars represent 10 μm. (**D**) qRT-PCR analysis of Ecad^Ncad/Δ^ EpiSCs in comparison to controls (vehicle) that show a general reduction in BMP signaling components. Expression was normalized to TBP and represented relative to controls (vehicle-treated). (**E**) Immunofluorescence staining of 4-OHT-treated and untreated Ncadki EpiSCs highlighting a general reduction in BMP signaling as activated mediators, the SMAD proteins, were less abundantly phosphorylated in recombined cells. N-cad is shown in red and pSMAD1/5/8 in green. Note, that N-cad is unexpectedly mainly localizing in the cytoplasm and nucleus. Scale bar represents 10 μm. (**F**) qRT-PCR analysis as in (**B**,**D**) of differentiation markers and key genes, acting downstream of BMP in the embryo. Levels are represented relative to untreated Ncadki cells.

To overcome the *in vivo* limitation of small cell numbers and to dissect autocrine from paracrine signals, originating in the extraembryonic ectoderm and contributing to the mutant phenotype, we made use of *in vitro* cultured epiblast stem cells (EpiSCs). We isolated EpiSCs carrying the Ncadki and Ecadflox alleles and stably transfected them with a tamoxifen-inducible Cre recombinase expression vector (pCAGGS-CreERT2)^36^. Upon E-cad depletion by tamoxifen treatment for three days Ecad^Ncad/Δ^ cells only slightly rounded up and partially lost their cell-cell contacts (Fig. 5A, Ecad^Ncad/fl^, +4-OHT), whereas this was not observed in untreated EpiSCs (Fig. 5A, Ecad^Ncad/fl^, vehicle). Conversely, Ecad^−/Δ^ cells showed a more drastic loss of cell-cell contacts combined with initial signs of differentiation by single cells emerging and acquiring a more mesenchymal shape (Fig. 5A, Ecad^−/fl^, +4-OHT). The lack of E-cad and the ectopic cadherin switching affected only mildly the primed pluripotent state of the isolated EpiSCs detected by unaffected *Oct4* expression and slight but significant reduction of *Nanog* and *Sox2* levels (Fig. 5B,C).

Ecad^Ncad/Δ^ cells displayed a significant downregulation of *Bmp4* transcripts and of the *bona fide* targets *Id2* and *Id3* (Fig. 5D and data not shown), combined with a reduction in pSmad1/5/8 immunoreactivity (Fig. 5E). The main receptor for Bmp4 in the epiblast, encoded by *Bmpr1a*, also showed a slight but not significant reduction upon E-cad depletion. Simultaneously, the intracellular effectors that counteract TGFβ signaling, like *Smad3* were less abundant in Ecad^Ncad/Δ^ cells (Fig. 5F), similar to the expression of mesoderm-specific genes, like *T-bra*, whereas *Nodal* was increased (Fig. 5F). *Smad5* expression was not affected, indicating that changes in BMP signaling were not due to reduced expression of downstream mediators of signal transduction. Although *Bmp4* is expressed at only low levels in the embryo^37^ robust expression was detected in all epiblast derived EpiSCs lines. The changes were specific for the induced cadherin switch as siRNA-mediated knockdown of E-cad alone was not altering BMP expression and signaling (Fig. S5C–E). Interestingly, reduction of increased *Nodal* expression in Ncadki EpiSCs by siRNA partially rescued the changes in *Bmp2* and *Bmp4* expression although the *Nodal* knockdown was very inefficient and siNodal Ctrl EpiSCs showed also increasing *Bmp2* and *Bmp4* expression Fig. S5F).

Our data show that a cell autonomous mechanism is active in the mouse epiblast by which N-cad expression enables modulation of cell-cell contacts and sustains pluripotency but also induces a specific differentiation process that is independent of the absence of E-cad. Downregulating of BMP signaling in Ecad^Ncad/Δ^ cells may be a direct or indirect consequence of altered cadherin expression and correlates with downregulation of TGFβ and WNT signaling that subsequently affects *T-bra* expression (Fig. 5F). These effects are in agreement with the observations in the embryo where blocking of signaling between the epiblast and the EXE is resulting in the mutant phenotype.

## Discussion

Cadherins are key mediators of cell sorting and tissue morphogenesis by providing selective and dynamic cell-cell adhesion. Several findings indicate that they are also crucial for regulating signaling, e.g. of the Igfr, Lifr, Egfr and Fgfr pathways^7^,^8^,^10^,^11^. We analyzed how cadherins are integrated into signaling cues in the postimplantation embryo and whether the cadherin switch, as a key event during EMT in the embryo and in cancer, is contributing to morphogenesis and the EMT program by modulating signaling pathways. Ectopic E-cad to N-cad switching in the mouse epiblast before gastrulation partially rescued the defects of an *E-cad* loss of function mutation, but resulted in severe defects at around E7.5 with a major reduction of the embryonic part. Despite severe morphological changes after E6.0 that manifested in a gradual loss of the characteristic cup-shape, PS and anterior-posterior (A-P) axis, mutant embryos successfully initiated gastrulation, but formed only a limited number of T-bra positive mesoderm cells and the amnion was absent. Ectopic cadherin switching resulted also in cell detachment from the epiblast and a reduction in basal lamina proteins as an indication of premature EMT. Molecularly, all defects were mainly attributed to impaired BMP signaling by reduced *Bmp4* transcription and activation of the downstream key factors *Cerl, Otx2* and *Wnt3a*, combined with an expansion of the *Nodal* expression domain.

The main function of cadherins is to establish cell-cell adhesion. Lack of E-cad results in defects in trophectoderm integrity of the blastocyst, in mammary gland, placenta and skin homeostasis^1^,^2^,^3^,^4^,^38^. In the small intestine and the trophectoderm the cell adhesion function of E-cad can be replaced by N-cad, a molecule that is widely absent in epithelia, but signaling defects are not rescued^8^,^39^. In line with these results, the defects in our Ncadki mutants were likely not a result of improper cell adhesion. By TEM and immunofluorescence labeling we detected formation of proper cell-cell junctions and maintenance of apical-basal cell polarity, indicating that structural requirements to initiate gastrulation and to form the amniotic fold and amnion were preserved. This is in contrast to the loss of *E-cad* e.g. in the skin that results in loss of cell polarity and break-down of the epithelial barrier^4^. Moreover, cell detachment was not seen in Ecadnull embryos, indicating a specific N-cad effect connected to its signaling function as observed in other systems^8^,^39^. Nevertheless, we cannot fully exclude that the introduction of an inappropriate cadherin molecule in the epiblast had opposite effects and artificially increased cell adhesion, even though reduced membrane-bound β-cat in our mutants was not in favor of this possibility.

Our data suggest that cadherin switching is leading to defects in BMP signaling that includes improper paracrine feedback to the EXE resulting in reduced *Bmp4* and *Bmp2* levels. In the normal embryo a very tight spatio-temporal control of *Bmp4*, Wnts, *Nodal, Cerl, Lefty1* and *Dkk1* in crosstalk between the epiblast, the AVE and the EXE is required to properly shape the embryo^40^. These genes help to correctly position the PS by inducing the expression of *Eomes, Lhx1, Foxa2, Otx2, Hesx1, Wnt3* and others in the PS region^40^. In our mutants mainly the restriction of *Nodal* expression to the proximal and posterior epiblast and proper rotation of the P-D into the A-P axis is lost or severely affected. Instead it is more uniformly distributed due to the reduced expression of *Cerl* and maybe of other Nodal inhibitors. Likely, the imbalance of the entire Bmp4/Nodal/Wnt3 reciprocal feedback loop results in inefficient repositioning of AVE markers, like *Otx2*. Interestingly, the expression domain of *Eomes*, the downstream effector of Nodal, is enlarged as a consequence of an increased effector domain. Our mutants phenocopy several aspects of BMP signaling loss-of function mutations including those of *Bmp4, Bmp2, Bmpr1a, Alk4* or *Nodal*^30^,^32^,^41^,^42^,^43^,^44^. The defects in these mutants also include improper amnion formation, e.g. in *Bmpr1a*-MORE and *Bmp2*^*−/−*^ embryos^30^,^37^. It was proposed that BMP signaling in the epiblast is required for proper recruitment of the prospective paraxial mesoderm (PXM) and in the *Bmpr1a*-MORE embryos mesoderm cells fail to respond to BMP signals. This results in abnormal morphogenetic movements and medial accumulation of these cells which is incompatible with PXM formation^37^. Our results indicate that upon ectopic cadherin switching the crosstalk between the epiblast and EXE that requires E-cad or is blocked by N-cad is compromised and leads to a reduction in BMP signaling with similar effects. In this scenario E-cad seems to be dominant over N-cad as E-cad^Ncad/+^ control mice do not show defects in BMP signaling and develop normally until adulthood^2^.

Several analyses indicate a correlation and crosstalk between E-cad and N-cad expression and BMP signaling in physiological contexts. During the neurulation of chicken embryos BMP is modulating N-cad levels in the perspective neurectoderm^45^. Bmp4 induces N-cad cleavage and cytosolic N-cad fragments block CBP from entering the nucleus^46^. In embryonic stem cells ectopic neural and mesenchyme induction is blocked by high levels of E-cad stabilized by BMP signaling^47^. It is tempting to speculate that these induced changes in cadherin levels and function in different systems are creating feedback loops to modulate BMP signaling as well. Indeed, such regulatory loops have been identified, e.g. during EMT processes of neural crest delamination. Here, BMP signaling is blocking N-cad and inducing cadherin-6B expression in pre-migratory neural crest cells, whereas N-cad inhibits de-epithelialization in the neuroepithelium by blocking BMP signaling^48^. These regulatory loops are required for proper morphogenesis and a similar mechanism is maybe active during mouse gastrulation that becomes deregulated in Ncadki mutants.

The observation of detachment or delamination of epiblast cells into the proamniotic cavity upon cadherin switching raises the question whether this is an indication of an ectopic EMT. It is possible that the ectopic cadherin switch in the epiblast already primes the cells to become motile and get ready for delamination into the primitive streak. In normal embryos this process is happening at the same time when gastrulation is initiated and the basement membrane is degraded. As in our mutants at the time-point of cadherin switching the basement membrane is still intact, the cells can only migrate into the direction of the least resistance and mechanical restrictions which likely is towards the proamniotic cavity. In addition, the reduced expression of *Cerl* is maybe resulting in inefficient protection of the AVE and favors ectopic induction of mesoderm. Interestingly, the delaminating cells are not (yet) expressing T-bra, which questions the identity of these putative EMT cells. Based on EM images the process of migration was not done in single cells as they were found in chains, still connected to the epiblast layer via the cell at the basis (Fig. S2D,E). This is reflecting the normal mesoderm migration process when the mesoderm is forming also with loose cell-cell contacts. This is supporting the idea of the induction of a premature EMT, occurring before the proper breakdown of the basal lamina, resulting in inappropriate migration.

Our results show that adhesion plays a pivotal role in controlling cell fate and morphogenetic movements to orchestrate embryonic shape and growth during the initial phases of postimplantation development. Cadherins are integral components of protein complexes to transmit signaling cues inside the cell. They are involved in progression of EMT in cells in which the program was already initiated, like at the onset of gastrulation. The dynamics of cell-cell adhesion and the interaction of cadherins with signaling receptors are regulating downstream signaling activities. This facilitates target gene expression and maintains feedback loops to reinforce established expression domains of key ligands and modulators. The analysis provides evidence that E-cad is also essential to maintain morphogen signaling in the epiblast during axis formation, indicating that a tight spatio-temporal control of cadherin expression is mandatory for morphogenesis independent of their function in tissue sorting.

## Methods

### Ethics statement

Animals were kept on a 12:12 h light-dark cycle and provided with food and water ad libitum. Animal husbandry and all experiments were performed according to the European Animal Welfare laws and guidelines. The protocols were approved by the committee on ethics of animal experiments of the state Baden-Württemberg (Regierungspräsidium Freiburg).

### Mice

Sox2Cre (Tg(Sox2-cre)1Amc), Ecadflox (Cdh1^tm2Kem^), Ncadki (Cdh1^tm4(Cdh2)Kem^) and R26R (Gt(ROSA)26Sor^tm1Sor^) mice were described elsewhere^2^,^3^,^17^,^39^,^49^. To generate cadherin switch embryos, Ecad^Ncad/ΔEpi^;Sox2Cre (Ncadki), timed matings with Ecad^Ncad/+^;Sox2Cre males and Ecad^flox/flox^ females were carried out. Ecad^−/ΔEpi^;Sox2Cre (Ecadnull) embryos were obtained in a similar way using Ecad^−/+^;Sox2Cre males. Embryos were isolated at the indicated embryonic day (E) after a vaginal plug was detected and fixed in 4% PFA for 2 h to overnight or processed directly.

### Embryo explant culture

6-well plates were incubated with Matrigel solution overnight at RT. Individually dissected epiblasts from E7.0 embryos were allowed to attach to a matrigel-covered well containing medium without FBS. Explants were kept in a humidified 37 °C incubator for 15 days changing the medium every other day and analyzed regularly with a brightfield stereomicroscope.

### Kidney capsule transplantation and teratoma formation

Embryos were dissected at E7.0, removing all extraembryonic tissues using a fine scalpel. Before transplantation, the syngenic recipient mouse was anaesthetized and the abdomen opened at the back at the level of the midline region between the end of the rib cage and the pelvis to expose the left kidney with a 1.5 cm longitudinal cut. The kidney was held in place with Desmarres chalazion forceps and a <1 mm cut was made into the air-dried kidney capsule. An isolated embryo was placed 3–4 mm under the lifted kidney capsule using a braking pipette^50^. The kidney was repositioned into the abdominal cavity and the surgical incision sealed with wound clips. Tumors were allowed to grow for 8 weeks and then isolated from the kidney, washed 2× in PBS and fixed in 4% PFA overnight.

### Whole mount *in situ* hybridization (WISH)

WISH was carried out as described previously^51^. In brief, 2-h fixed embryos were permeabilized by a methanol:phosphate-buffered saline/0.1% Tween (PBT) dehydration series and treated with 10 μg/ml proteinase K in DEPC treated PBT at RT. After post-fixation and washing in PBT hybridization solution (50% formamide, 5× SSC pH 4.5, 0.1% Tween, 50 μg/ml Heparin, 50 μg/ml tRNA) was applied and incubated at least 1 h at 70 °C before 1 μg/ml DIG-labeled RNA probe was added and incubation continued at 68 °C overnight. The hybridization solution was removed, embryos rinsed 4× for 30 min with pre-warmed hybridization solution at 65 °C, followed by 2× PBT and 3× maleic acid buffer/0.1% Tween (MABT) for 30 min each. After blocking in 1× MABT/1% Blocking reagent (Roche)/10% heat inactivated sheep serum the embryos were incubated with anti-DIG alkaline phosphatase-conjugated antibody at 1:2000 in MABT overnight at 4 °C. Unspecific antibody binding was eliminated by 4× hourly washes in MABT followed by 3× washes for 20 min each in PBS pH9.5. Color reaction was carried out in BM Purple AP substrate (Roche) and stopped by washing embryos in PBT.

### TUNEL staining

TUNEL staining of dissected embryos was performed using *In Situ* Cell Death Detection Kit (Roche) according to the manufacturer’s instructions. Imaging and orthogonal projections were performed with a Zeiss Spinning disc microscope and ZEN software (Zeiss).

### Paraffin embedding and sectioning

PFA-fixed embryos and teratomas were washed in PBS. After dehydration in Ethanol/PBS and Histolemon series they were incubated in paraffin at 58 °C overnight and casted into molds covered with paraffin. Blocks were sectioned using an RM2155 microtome (Leica) at 5–7 μm.

### Hematoxylin and Eosin (H&E) staining

Paraffin sections were dewaxed and rehydrated step-wise. The sample were stained in hematoxylin solution (0.1% hematoxylin, 5% KAl(SO_4_)_2_, 0.02% KIO_3_). Counterstaining was performed by incubating the slides in eosin solution (1% eosin). Slides were mounted in Permount Mounting Medium (Fisher Scientific) and analyzed using brightfield microscopy.

### Immunohistochemistry (IHC) and immunofluorescence (IF) stainings

After rehydration of slides, endogenous peroxidase activity was blocked by incubation in 1% H_2_O_2_/0.42% citric acid/1.08% Na_2_HPO_4_) and then boiled at 95 ±1 °C for 20 min in 40 mM Tris-HCl/64 mM EDTA pH9.0. Specimens were incubated in 1% BSA/PBS for 30 min followed by a 1-h to overnight incubation with the primary antibody in 0.05% BSA/PBS. After washing the slides briefly 2× in PBS and 1× in PBS-T (PBS/0.2% Tween) the samples were incubated for 45 min with EnVision+ (mouse or rabbit, DAKO) followed by color development in DAB substrate (Sigma) and mounted in Kaiser’s Glycerin-Gelatin for IHC. Incubation with Alexa488 and Alexa594-conjugated secondary antibodies was carried out for IF and slides were mounted with Citifluor AF1 antifadent solution (Citifluor limited)/DAPI.

Cells were plated on gelatin-coated cover slips for IF. After treatment they were washed in ice-cold PBS and fixed by either methanol or PFA treatment, −20 °C methanol for 15 min was used for membrane proteins and 4% PFA for 10 min followed by a 0.5% Triton-X100/PBS permeabilization step for 5 min for nuclear proteins. After blocking cells in 1% BSA they were incubated overnight in primary antibody solution diluted 1:200 in PBS. Secondary antibody incubation and mounting was done as described above. All IF samples were analyzed by confocal microscopy using a Zeiss Spinning disc microscope.

### Scanning and transmission electron microscopy

Embryos were fixed in 2% glutaraldehyde/1% PFA in 0.1 M phosphate buffer pH7.3 and in 1% osmiumtetroxide for 1 h, followed by washes in H_2_O. Embedding for transmission electron microscopy (TEM) continued with dehydration through an increasing acetone series and infiltration with epoxy resin (Epon 812, Serva, Heidelberg, Germany) before embryos were cured in rubber molds at 60 °C for 2 days. Resin blocks were trimmed with a trimming mill (Leica EM Trim 2, Leica Microsystems, Wetzlar, Germany) prior to microtome sectioning (Ultracut S, Reichert, Wien, Austria) with a diamond knife (Diatome, Biel, Switzerland). Selected semithin sections (approximately 0.5 μm) were stained with toluidine blue and subsequent thin sections (50–70 nm) were transferred to copper grids and stained with uranyl acetate and lead citrate and were examined on a Zeiss EM902 electron microscope operated at 80 kV (Carl Zeiss, Oberkochen, Germany). Digital images were acquired with a side-mounted 1 × 2k FT-CCD Camera (Proscan, Scheuring, Germany) using iTEM camera control and imaging software (Olympus Soft Imaging Solutions, Münster, Germany).

For scanning electron microscopy (SEM) after osmiumtetroxide treatment for 45 min embryos were dehydrated with a graded series of ethanol. Subsequent critical point drying by immersion in CO_2_ was performed in a Emitech K850 critical point dryer (Emitech Ltd. Ashford, UK). Dried embryos were dissected carefully on parafilm coated glass petri dishes and embryonic halves were mounted on SEM stubs with adhesive carbon tape (Plano, Wetzlar, Germany) and sputter-coated with a gold layer of approximately 15–20 nm thickness (Bal-Tec SCD004, Balzers Union Ltd., Balzers, Liechtenstein). Specimens were viewed on a Zeiss DSM960A SEM equipped with digital image scanning and processing systems and software (Point Electric, Halle, Germany) operated at 10 kV and in a field-emission SEM Zeiss Merlin VP compact operated at 5 kV.

### Isolation and culture of epiblast stem cell (EpiSCs)

E6.0–6.5 embryos were dissected in FHM medium (Millipore) and the epiblast was isolated as described^52^. Briefly, the embryonic portions were collected separately and grown in CDM (F12 Nutrient Mix/IDMEM, 5 mg/ml BSA, 450 μM Monothioglycerol, 7 μg/ml Insulin (Sigma), 15 μg/ml Transferrin (Roche), 100 IU/ml Pen/Strep, 1% Chemically Defined Lipid (Life Technologies), 12 ng/ml bFGF (R&D Systems), 20 ng/ml Activin A (Peprotech), 10 μM ROCK Inhibitor (Y27632, Tocris) at 37 °C and 6% CO_2_. 3–4 days after plating individual epiblast outgrowths were partially dissociated into smaller pieces using a scalpel and transferred into freshly gelatin/DMEM/15% FCS-coated wells. After one week EpiSC colonies became visible, they were picked individually and processed as above. From the second passage onward the colonies were separated by 1mg/ml Collagenase IV (Invitrogen) treatment for two minutes at RT. After washing they were transferred into new wells, with special care to maintain big cell clusters. The ROCK inhibitor improved proper attachment of the colonies to the plates^53^. After long-term passaging the established EpiSCs stabilized in a more homogenous cell population.

### Transfection of siRNA and plasmid DNA

siRNAs (Supplementary Table S2) and plasmid DNA was transfected into EpiSCs using the Fugene6 reagent (Promega). Equal numbers of cells were seeded on 6-well plates and transfected within 4 h with the different siRNAs (100 pmol per well) or plasmid DNA (4 μg per well). siRNAs, plasmids and transfection reagent were diluted in CDM. Cells were harvested 36 h or 96 h post-transfection. Cells were stably transfected with the Cre recombinase expression vector pCAGGS-CreERT2^36^. Induction of recombination was carried out by adding 0.1 μg/ml Tamoxifen to the medium for three days.

### Quantitative reverse transcriptase-PCR (qRT-PCR) expression analysis

Total RNA was isolated using the NucleoSpin RNAII kit (Macherey-Nagel) according to the manufacturer’s protocol. qRT-PCR was performed using the ABsolute QPCR ROX Mix (Thermo Scientific) together with the mouse Universal Probe Library (Roche) according to the manufacturer’s instructions. Primers and Universal Probes are given in Supplementary Table S1. Experiments were done in technical and biological triplicates using a 7300 Real Time PCR System (Applied Biosystems).

### Western blot analysis

For preparation of whole cell-lysates cells were harvested by scraping in PBS containing Ca^2+^ and Mg^2+^ and lysed in Golden Lysis Buffer (20 mM Tris-HCl pH 7.9, 137 mM NaCl, 1% Triton, 10% Glycerol, 10 mM Na_3_OVa_4_ ,10 mM NaF, 1 mM Na-pyrophosphate, 1 mM PMSF, 1× complete protease inhibitor cocktail (Roche)). After sonication lysis was completed by incubation for 30 min at 4 °C gently shaking. After centrifugation at 14,000 rpm for 10 min the soluble fraction was used for further analysis. Western blot was performed as previously described^8^.

### Antibodies

The following antibodies were used: mouse anti-Oct4 (Santa Cruz), rabbit anti-Nanog^54^, anti-Sox2 (Calbiochem), rabbit anti-E-cad (extracellular)^55^, mouse anti-E-cad (intracellular, BD Bioscience), mouse anti-N-cad (BD Bioscience), mouse anti-N-cad (Life Technologies), rat anti-ZO-1^56^, rat anti-pan-Laminin^57^, goat anti-T-brachyury (Santa Cruz), goat anti-Sox17 (R&D Systems), rabbit anti-pH3 (Cell Signaling), rabbit anti-H3 (Abcam), mouse anti-β-catenin (BD Bioscience), mouse anti-Ezrin (Cell Signaling) and rabbit anti-pSMAD1-5-8 (Cell Signaling).

### Statistical analysis

Statistical analysis was performed using one-way, two-way ANOVA and unpaired t-test of the GraphPad Prism software where appropriate. Graphs are presented as mean ± SEM. Results were considered significant when the false discovery rate (p-value) was below 5%, *p < 0.05; **p < 0.01; ***p < 0.001, ns, not significant.

## Additional Information

**How to cite this article**: Basilicata, M. F. *et al*. Inappropriate cadherin switching in the mouse epiblast compromises proper signaling between the epiblast and the extraembryonic ectoderm during gastrulation. *Sci. Rep.* **6**, 26562; doi: 10.1038/srep26562 (2016).

## Supplementary Material

Supplementary Information
