# Supplementary material for: Inappropriate cadherin switching in the mouse epiblast compromises proper signaling between the epiblast and the extraembryonic ectoderm during gastrulation
Source: Sci Rep. 2016 May 24;6:26562. doi: 10.1038/srep26562 (PMC4877576; doi:10.1038/srep26562)
Supplement: Supplementary Information [file srep26562-s1.pdf]

Inappropriate cadherin switching in the mouse epiblast compromises proper signaling between the epiblast and the extraembryonic ectoderm during gastrulation

by

M. Felicia Basilicata, Marcus Frank, Davor Solter, Thomas Brabletz, and Marc P. Stemmler

Supplementary Information

Supplementary Figures S1-S5
Supplementary Tables S1, S2

Figure S1

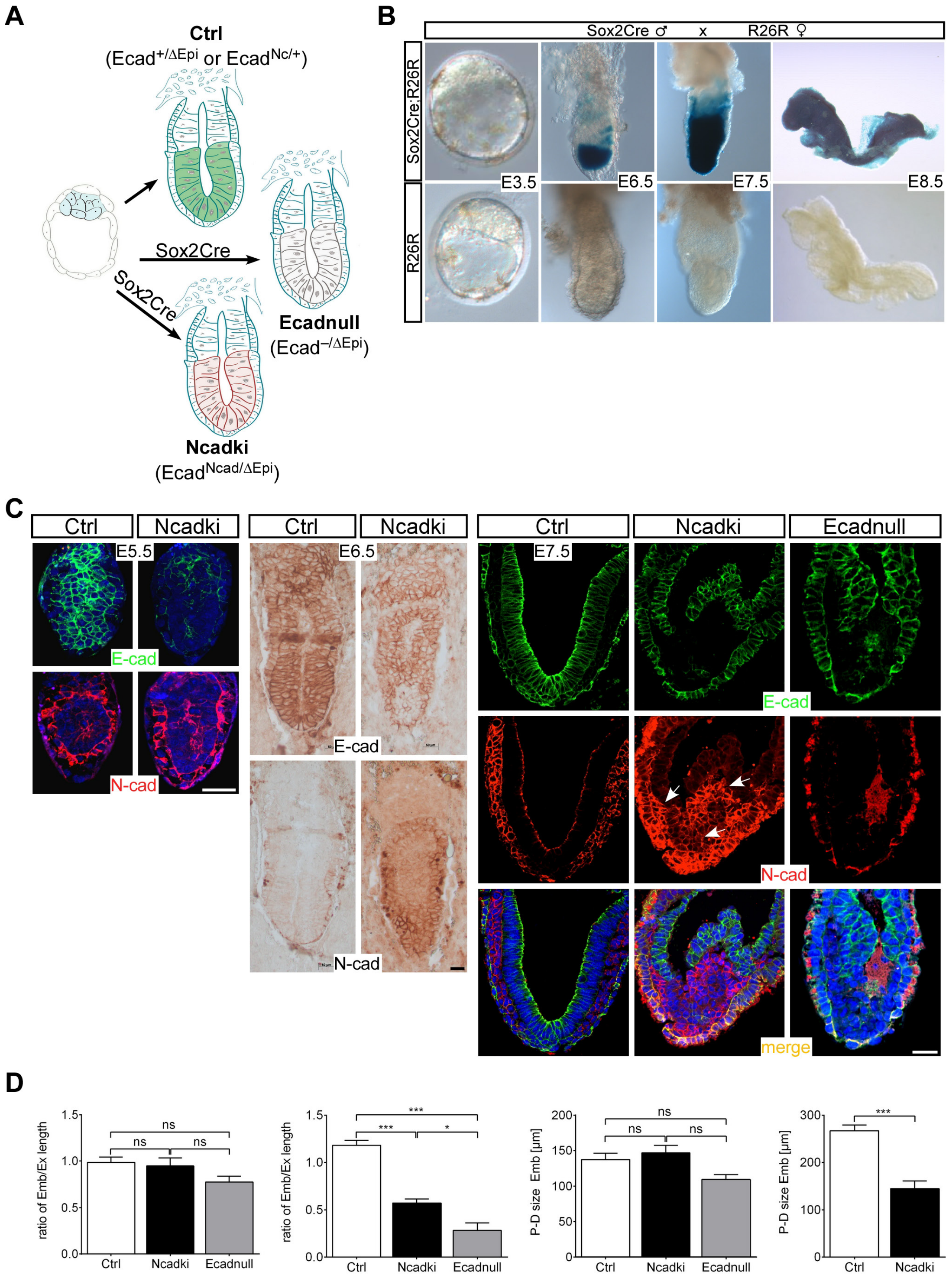
A
Ctrl
(Ecad+/ΔEpi or EcadNc/+)
Sox2Cre
Sox2Cre
Ecadnull
(Ecad−/ΔEpi)
Ncadki
(EcadNcad/ΔEpi)
B
Sox2Cre ♂ x R26R ♀
Sox2Cre;R26R
R26R
E3.5
E6.5
E7.5
E8.5
C
Ctrl
Ncadki
E5.5
E-cad
N-cad
Ctrl
Ncadki
E6.5
E-cad
N-cad
Ctrl
Ncadki
Ecadnull
E7.5
E-cad
N-cad
merge
D
ratio of Emb/Ex length
ns
ns
ns
Ctrl
Ncadki
Ecadnull
ratio of Emb/Ex length
***
***
*
Ctrl
Ncadki
Ecadnull
P-D size Emb [µm]
ns
ns
ns
Ctrl
Ncadki
Ecadnull
P-D size Emb [µm]
***
Ctrl
Ncadki

Figure S1: Detailed analysis of Sox2Cre recombination efficiency and of the Ecad$^{Ncad/\Delta Epi}$ phenotype. (A) Schematic representation of the induced cadherin switch. Sox2Cre becomes activated after the blastocyst stage in the epiblast (green). Its activity depletes E-cad from the epiblast after implantation (Ecadnull, grey cells) and induces the cadherin switch in Ecad$^{Ncad/\Delta Epi}$ with exclusively N-cad expression (Ncadki, red), whereas the control epiblast remains E-cad+ (Ctrls, green). (B) Analysis of Sox2Cre recombination efficiency in a spatio-temporal manner using the R26R allele between E3.5 and E8.5, displaying complete recombination already in E6.5 epiblasts. (C) Analysis of recombination efficiency of Ecad^{fl} in Ncadki and Ecadnull embryos by immunofluorescence (left and right panels) and immunohistochemistry staining (middle panel) at E5.5 (left), E6.5 (middle) and E7.5 (right). Already at E5.5 E-cad is properly depleted and only in a few embryos progeny of unrecombined escapers is detected. Arrows indicate variability in the N-cad expression levels between mesoderm (endogenous N-cad) and Ncadki expression in the epiblast. Scale bars represent 50 µm. (D) Quantification of the proximal-distal (P-D) extension of the epiblast in Ncadki and Ecadnull embryos (n=4-14). The ratio of the extraembryonic (Ex) and the embryonic (Emb) is shown and absolute sizes of the epiblasts at E6.5 and E7.5 are given. Embryos are presented with proximal to the top, anterior to the left (E6.5-E7.5) or anterior to the left and dorsal to the top (E8.5).

Figure S2

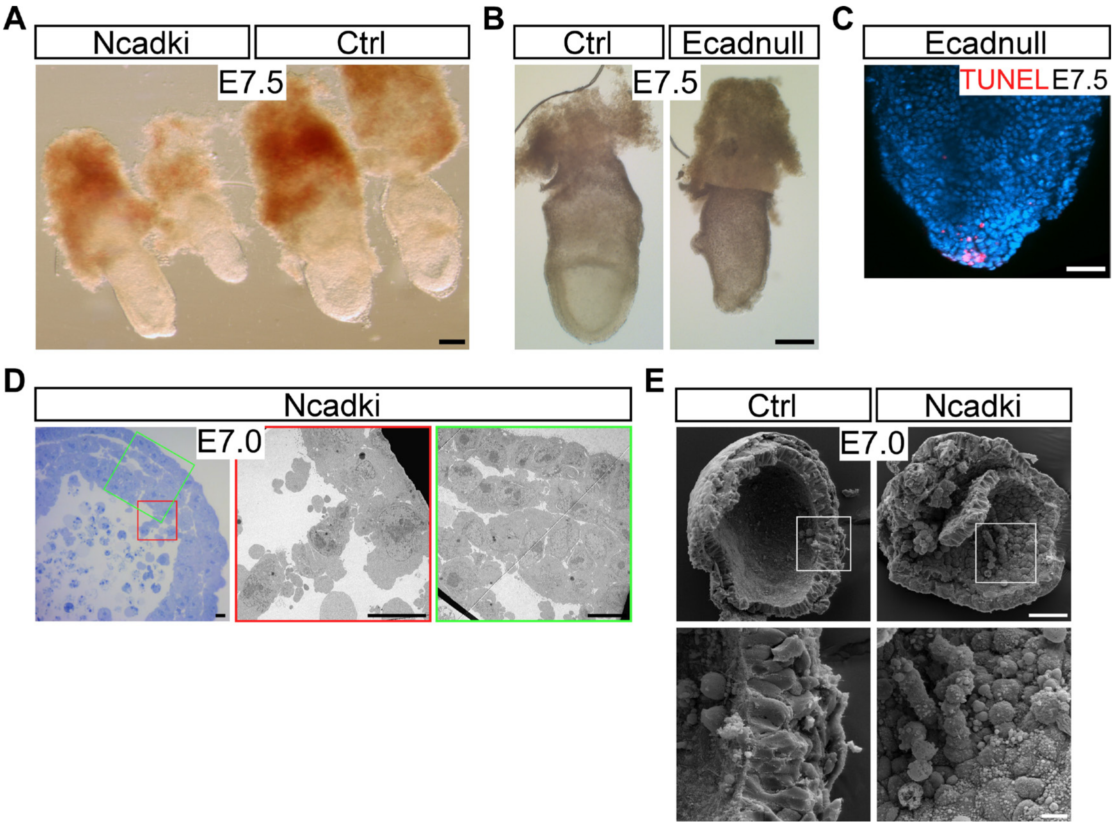

Figure S2: E-cad is efficiently depleted in Ecad$^{Ncad/\Delta Epi}$ embryos at E5.5. (A) Variability in the mutant phenotype observed at E7.5, mainly characterized by size differences of the epiblast of mutants. Scale bar represents 100 µm. (B, C) Morphological examination (B) and TUNEL staining (C) of Ecadnull embryos at E7.0, showing increased apoptosis in absence of adhesion in the epiblast. Scale bars represent 100 µm for bright field images and 50 µm for TUNEL. (D) Semithin and TEM images of mutant and control embryos, visualizing the cell detachment process in the mutants. Scale bars represent 10µm. (E) Ultrastructural analysis by scanning electron microscopy shows epiblast cells detaching from epiblast in chains in Ncadki E7.0 embryos. Scale bars represent 50 µm for upper and 10 µm for lower panels.

Figure S3

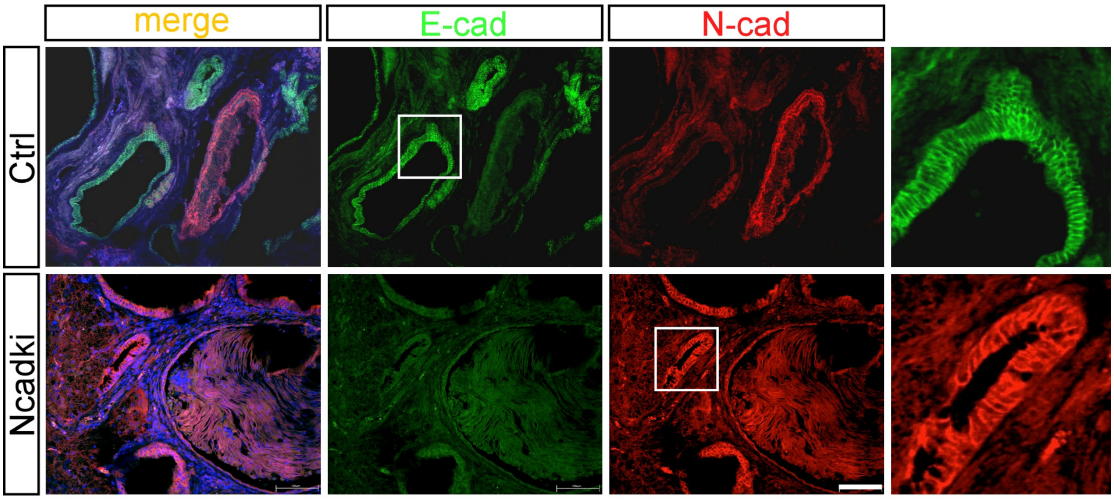

Figure S3: Teratomas of Ncadki embryos show complete recombination in areas with a large variety of different tissues. Cells that have differentiated into epithelia (keratinized epidermis-like cysts and enterocyte and goblet cell-containing intestinal tissues) are E-cad+ in control specimen but exclusively express N-cad in mutatnt specimen. No E-cad+ cell was detected in Ncadki-derived teratomas. Scale bar represents 100 µm.

Figure S4

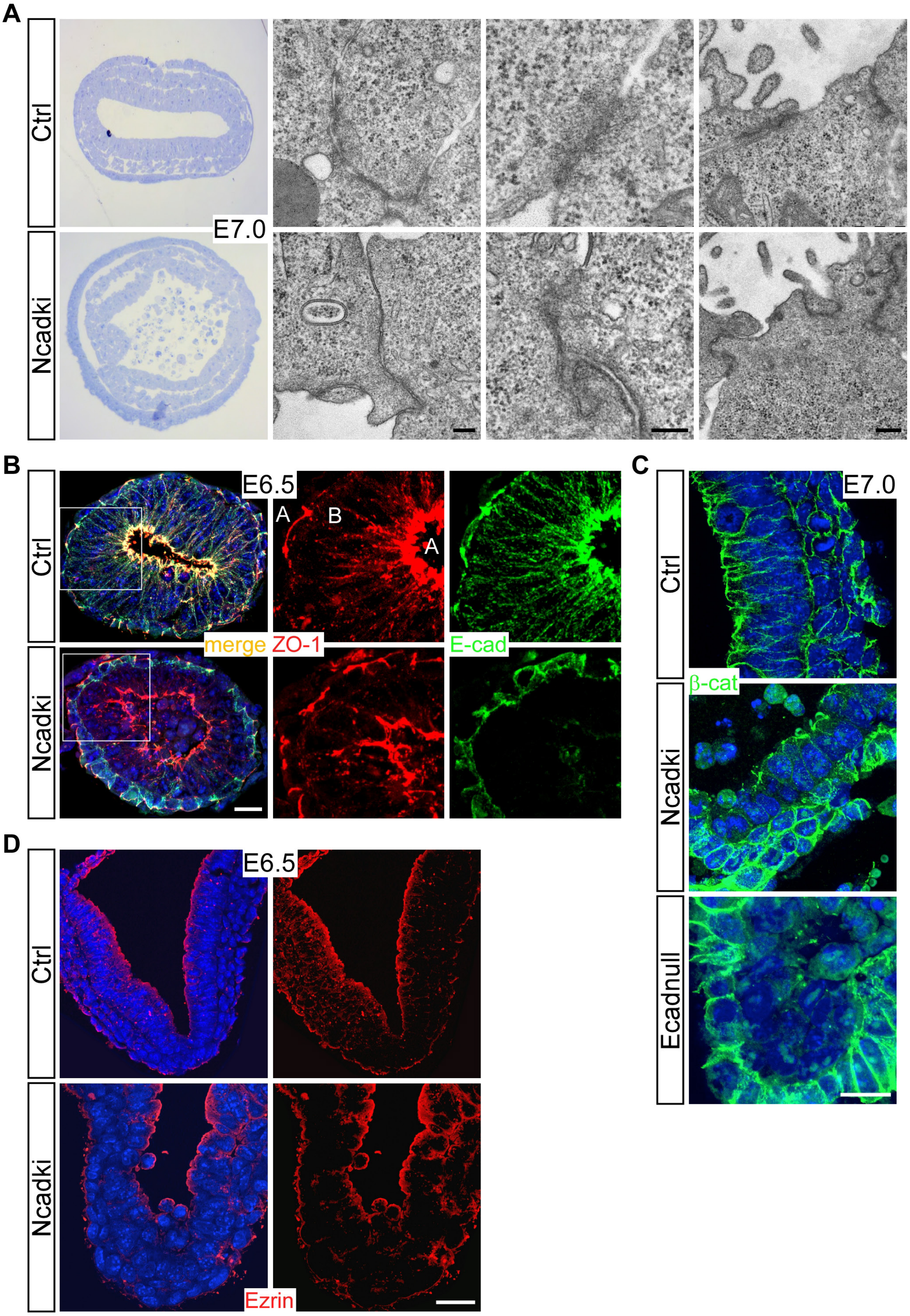
A
Ctrl
Ncadki
E7.0
B
Ctrl
Ncadki
E6.5
A
B
A
merge
ZO-1
E-cad
C
E7.0
Ctrl
β-cat
Ncadki
Ecadnull
D
E6.5
Ctrl
Ncadki
Ezrin

Figure S4: Ncadki embryos form normal adherens junctions and cells of the epiblast show proper apical-basal polarity. (A) Semithin sections and TEM analysis of control (upper) and Ncadki embryos (lower), showing proper formation of adherens and tight junctions in mutant epiblast cells. Note, that apical villi are forming in Ncadki embryos similar to controls. Scale bars represent 200 nm. (B) Immunofluorescence staining of E-cad (green) and the tight junctional protein ZO-1 (red). Similar to controls ZO-1 is concentrated at the apical side and E-cad is absent from the epiblast of Ncadki embryos. A, apical; B, basal. Scale bar represents 20 µm. (C) Confocal images of immunofluorescently labeled sections of Ncadki and control embryos with anti-β-catenin antibody (green), showing proper distribution of adherens junctions and adherens junction assembly, but a decreased intensity in Ncadki in comparison to control embryos. No membrane staining is detected in Ecadnull epiblasts. Scale bar represents 10 µm. (D) Immunofluorescence labeling of the apical marker Ezrin (red) confirms proper apical-basal cell polarity in E7.5 Ncadki epiblast cells. Nuclei are stained with DAPI. Scale bars represent 50µm.

Figure S5

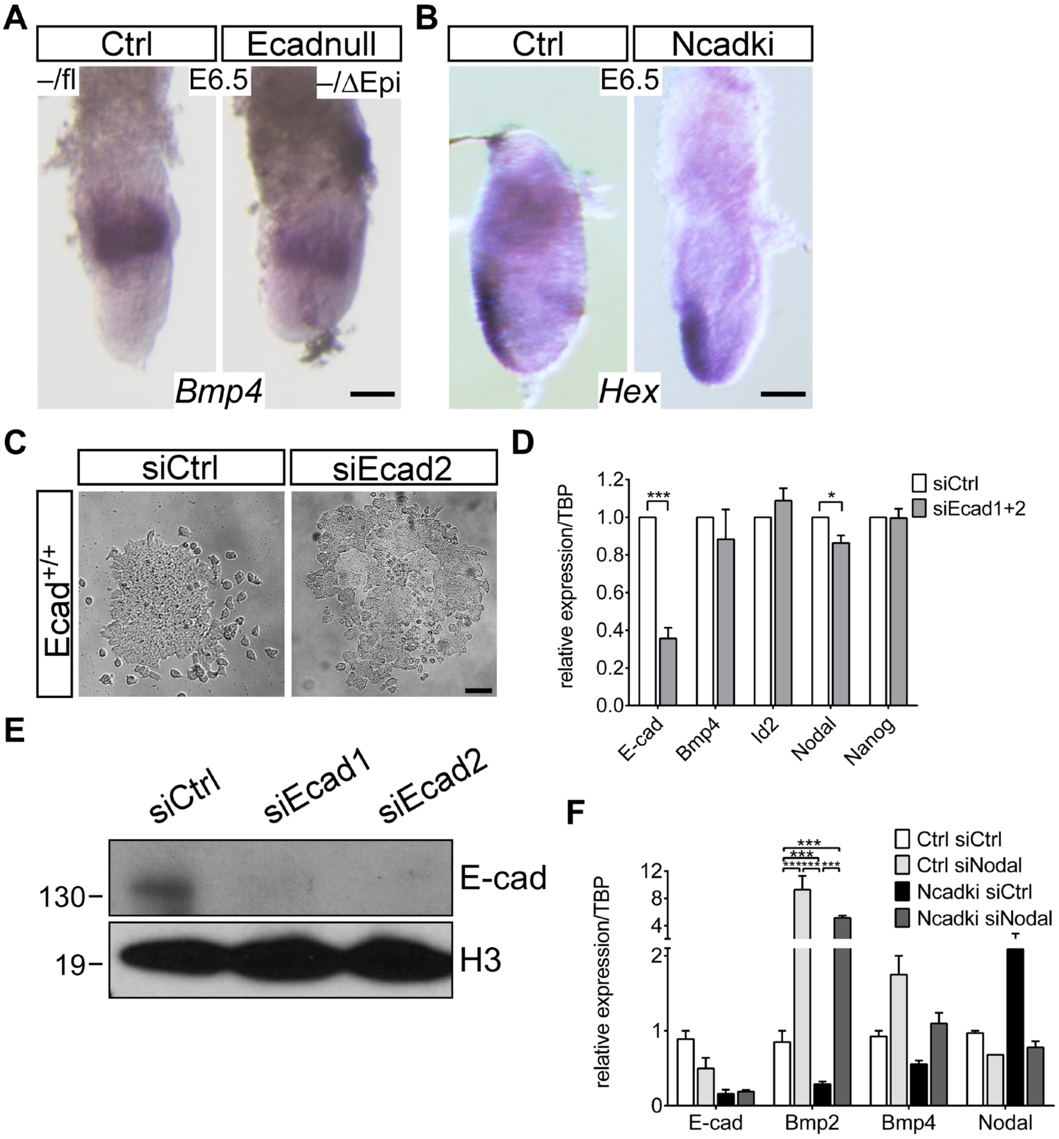
A
Ctrl
Ecadnull
–/fl
E6.5
–/ΔEpi
Bmp4
B
Ctrl
Ncadki
E6.5
Hex
C
siCtrl
siEcad2
Ecad+/+
D
relative expression/TBP
siCtrl
siEcad1+2
E-cad
Bmp4
Id2
Nodal
Nanog
E
siCtrl
siEcad1
siEcad2
130
19
E-cad
H3
F
relative expression/TBP
Ctrl siCtrl
Ctrl siNodal
Ncadki siCtrl
Ncadki siNodal
E-cad
Bmp2
Bmp4
Nodal

Figure S5: Loss of E-cad alone is not accounting for reduction of BMP signaling in EpiSCs. (A) Whole mount *in situ* hybridization of Bmp4 in $Ecad^{-/fl}$ (Ctrl) and $Ecad^{-/\Delta Epi}$ (Ecadnull) E6.5 embryos showing that mono- and biallelelic E-cad depletion is not substantially affecting BMP signaling. Scale bar represents 100 µm. (B) Analysis of Hex expression in control and Ncadki E6.5 mutant embryos. Scale bar represents 100 µm. (C) Cell morphology of $Ecad^{-/fl}$ cells after siRNA-mediated E-cad knockdown (siEcad). Cells lose cell-cell contacts and become scattered as single cells. Scale bar represents 50 µm. (D) Western blot analysis of whole-cell lysates upon E-cad siRNA-mediated knockdown showing efficient E-cad depletion with two individual siRNAs using the antibody detecting the extracellular domain (extra-) of E-cad. Analysis of histone H3 (H3) is shown to verify equal loading. (E) qRT-PCR analysis of a subset of genes used in Fig. 5D, F upon E-cad knockdown with a mixture of two different siRNAs, showing that E-cad depletion alone is not altering BMP signaling in EpiSCs. (F) qRT-PCR analysis of $Ecad^{Ncad/\Delta}$ (Ncadki) and $Ecad^{+/\Delta}$ EpiSCs showing a partial rescue of Bmp expression upon Nodal depletion in Ncadki. Note, that the Nodal knockdown is very inefficient. Statistical significance was detected only for Bmp2 using 2-way ANOVA.

Table S1: Primers and UPL probes used for qRT-PCR

Gene	forward (5' → 3')	reverse (5' → 3')	UPL
Actb (β-Actin)	AAGGCCAACCGTGAAAAGAT	GTGGTACGACCAGAGGCATAC	56
Cdh1 (E-cad)	AGTGTTTGCTCGGCGTCT	GCAAAGCCATGAGGAGACC	18
Cdh2-ki (N-cad knockin allele)	CCATGGCCACTAGTATGTGC	AATTTCACCAGAAGCCTCCA	74
Nanog	TTCTTGCTTACAAGGGTCTGC	AGAGGAAGGGCGAGGAGA	110
Oct4	GTTGGAGAAGGTGGAACCAA	CTCCTTCTGCAGGGCTTTC	95
Sox2	TCCAAAAACTAATCACAACAATCG	GAAGTGCAATTGGGATGAAAA	63
Tbp	CGGTCGCGTCATTTTCTC	GGGTTATCTTCACACACCATGA	107
Cerl	CTGTGCCCCTTCAACCAGAC	AGCAGTGGGAGCAGAAGC	105
Fgf5	AAAACCTGGTGCACCCTAGA	CATCACATTCCCGAATTAGC	29
Bmp4	GGATCTTTACCGGCTCCAG	GGGATGTTCTCCAGATGTGTTCTT	21
Bmpr1a	TGCTTGAGATACTCTTACAATAATGCT	TGGCTGTCTGTATAGTTGCTATGAT	78
Nodal	CCAACCATGCCTACATCCA	CACAGCACGTGGAGGAAC	40
T (T-bra)	GCCAGCCCACCTACTGG	GAGCCTGGGGTGATGGTA	100
Id2	ACAGAACCAGGCGTCCAG	AGCYCAGAAGGGAATTCAGATG	89
Mmp7	TAATTGGCTTCGCAAGGAG	AGTGTTCCCTGGCCCATC	94
Itgb1 (CD29)	TGGCAACAATGAAGCTATCG	ATGTCGGGACCAGTAGGACA	109
Smad5	CAAGCTCTGGACCTGGAAGT	GAATTATCTGGGGCCATCTG	33
Smad3	GCCACTGTCTGCAAGATCC	AGCTAGGAGGGCAGCAAAT	64
Msx2	AGGAGCCCGGCAGATACT	GTTTCCTCAGGGTGCAGG	70
Ctnnb1 (β-cat)	GCTGACCAGTTCCCTCCTTCA	CACCAATGTCCAGTCCAAGA	21
Lefty1	ACTCAGTATGTGGCCCTGCT	AACCTGCCTGCCACCTC	76

Table S2: siRNAs used for knockdown experiments

siRNA/gene	Sequence or Catalogue Number	Provider
Mm_Cdh1_1	CTCGCGGATAACCAGAACAAA	Qiagen FlexiTube Gene solution _2
Mm_Cdh1_2	TACCATGTTTGCTGTATTCTA	Qiagen FlexiTube Gene solution_5
Mm_Cdh1 Mix		Qiagen FlexiTube Gene solution_2&5
Mm_Nodal	SI01328936, SI01328929, SI01328922, SI01328915	Qiagen FlexiTube Gene solution
negative control	ON-TARGET*plus* Non-targeting siRNA #1 Dharmacon, D-001810-01-05	Dharmacon
